# RELIGION AND LONELINESS: OLDER PEOPLE IN IRELAND

**DOI:** 10.1093/geroni/igae098.0630

**Published:** 2024-12-31

**Authors:** Gerard Leavey

**Affiliations:** Ulster University, Belfast, Northern Ireland, United Kingdom

## Abstract

**Background:**

Religious identity and adherence may confer health benefits, through increased social capital (eg, community belonging, and material resources) and mechanisms for coping with adversity and misfortune. Isolation and loneliness are associated with less engagement in religious practice and can influence mental health outcomes. Aims: To investigate comorbid depression (or subthreshold symptoms) in those with clinically relevant anxiety and their relationship with loneliness, social networks, religious practice and long-term illness.

**Methods:**

Secondary data analysis of Wave 1 of the TILDA study (2009-2011), a nationally representative community-based sample.

**Results:**

Compared to those with low/no symptoms of depression, those classed as comorbid were more likely to be male, had lower education levels, had spent more time abroad, lower religious attendance, a limited social network, were lonelier and had a long-term life-limiting illness. Those with subthreshold levels of depression reported a more restricted social network and more moderate levels of loneliness.

